# Decoding Abdominal Pain in Constipation-predominant Irritable Bowel Syndrome and Functional Constipation: Mechanisms and Managements

**DOI:** 10.1007/s11894-025-00967-7

**Published:** 2025-03-17

**Authors:** Jingyuan Luo, Qianqian Xu, Shujun Xu, Lixiang Zhai, Chun-Su Yuan, Zhaoxiang Bian

**Affiliations:** 1 Vincent V.C. Woo Chinese Medicine Clinical Research Institute, School of Chinese Medicine, Hong Kong Baptist University, 3/F, Jockey Club School of Chinese Medicine Building, 7 Baptist University Road, Kowloon Tong, Hong Kong, SAR China; 2 Center for Chinese Herbal Medicine Drug Development and School of Chinese Medicine, Hong Kong Baptist University, Hong Kong, SAR China; 3 Tang Center for Herbal Medicine Research and Department of Anesthesia and Critical Care, Pritzker School of Medicine, University of Chicago, 5841 South Maryland Avenue, MC 4028, Chicago, IL 60637 USA; 4 Department of Anesthesia and Critical Care, Pritzker School of Medicine, University of Chicago, Chicago, IL 60637 USA; 5 Department of Pharmaceutical Sciences, School of Pharmacy and Pharmaceutical Sciences, University at Buffalo, State University of New York, Buffalo, NY 14214-8033 USA

**Keywords:** Abdominal pain, IBS-C, FC, Mechanism, Management

## Abstract

**Purpose of Review:**

Abdominal pain in constipation-predominant irritable bowel syndrome (IBS-C) and functional constipation (FC) remains a difficult clinical challenge due to unclear pathophysiological mechanisms and limited pain-targeted treatments. This review critically evaluates the evidence on the underlying pain mechanisms in IBS-C and/or FC and explores management strategies, their limitations, and future directions.

**Recent Findings:**

Most research on constipation-related pain is based on IBS-C patients or animal models, with limited studies focusing on FC. Visceral hypersensitivity, serotonin dysregulation, gut-brain axis dysfunction, and central/peripheral nervous system alterations are implicated in IBS-C pain, while FC pain is less studied and may be primarily linked to colonic distension and motility dysfunction. Management strategies include 5-HT4 agonists, GC-C agonists, chloride channel activators, psychological therapies, probiotics and complementary medicine.

**Summary:**

Despite available treatment options, managing abdominal pain in IBS-C and FC remains challenging due to heterogeneous pathophysiology and limited targeted therapies. While some interventions provide symptomatic relief, there is no universally effective treatment for abdominal pain across all patients. Future research should focus on identifying pain-specific biomarkers, refining diagnostic criteria, and integrating multi-omics data and neuroimaging techniques to better distinguish pain mechanisms in IBS-C versus FC and develop more precise, patient-centered interventions.

## Introduction

Constipation is a prevailing condition featured by difficulties in defecation, including infrequent bowel movements, hard or lumpy stools, excessive straining, sensation of incomplete evacuation or blockage, and use of manual maneuvers to facilitate evacuation [1]. Once patients experience persistent symptoms for at least 3 months, they are considered as suffering from chronic constipation [1]. This condition is found in 14% worldwide, affecting all demographics, with higher prevalence in women, the elderly, and lower socioeconomic groups [2], making it the sixth most common gastrointestinal issue and causing a large social burden [3, 4].

Based on the presentation of symptoms, Rome criteria further categorize primary chronic constipation into functional constipation (FC) and constipation-predominant irritable bowel syndrome (IBS-C) [5]. According to the Rome IV criteria, FC is diagnosed when patients have chronic constipation symptoms without meeting IBS criteria and exhibit at least two of the following: < 3 spontaneous bowel movements (SBMs) per week, straining, incomplete evacuation, manual maneuvers to aid defecation, hard/lumpy stools, or anorectal obstruction/blockage in > 25% of defecations. IBS-C is characterized by recurrent abdominal pain (≥ 1 day/week), associated with at least two of the following: stool form changes (> 25% hard/lumpy and < 25% mushy/liquid), altered stool frequency, or pain related to defecation. A recent epidemiological study revealed that the worldwide prevalence of FC and IBS-C in adults is 11.7% and 1.3% respectively [2].

While recurrent abdominal pain and the temporal relationship of pain with defecation serve as the primary differentiating criteria between FC and IBS-C [6], the substantial overlap in symptoms suggests that these disorders may exist along a spectrum rather than as entirely distinct entities [5, 7]. According to studies of our own [8, 9] and those of others [10–15], abdominal pain is not exclusive to IBS-C and can also occur in a subset of patients with FC. The transition from FC to IBS-C, or vice versa, has also been reported [10]. These findings suggest that pain frequency alone may be insufficient for making a clear distinction between FC and IBS-C [13–15]. Despite their overlapping features, the underlying pathophysiological mechanisms of abdominal pain in FC remain poorly understood, and it is unclear whether the pain mechanisms in FC resemble those in IBS-C or arise from distinct processes [16, 17].

Abdominal pain, particularly in IBS-C, is a key driver of disease burden, significantly affecting psychological well-being, treatment outcomes, and quality of life [18–21]. The persistent and unpredictable nature of abdominal pain further complicates disease management and can negatively impact treatment adherence. In IBS-C, pain is considered a core diagnostic feature, whereas in FC, its presence is more variable and less well-characterized. Understanding the mechanisms underlying pain generation in both conditions is essential for improving diagnostic accuracy and optimizing treatment strategies.

Together, it should be noted that unknown mechanisms behind abdominal pain, the complex crosstalk between abdominal pain and the progression of constipation, as well as limited treatment approaches, making the management of abdominal pain in IBS-C and FC difficult. While some researchers have attempted to decipher the pathophysiological mechanism behind IBS-C and FC and its associated abdominal pain, no summative evidence has been synthesized, pointing to the urgency of comprehensive reviews of studies addressing these particular issues.

To facilitate understanding, we comprehensively review current literature on possible underlying pathophysiological mechanisms and treatment strategies for managing abdominal pain in constipation. Based on the summarized evidence, we also critically discuss the limitations and propose future directions that are expected to fill current research and clinical gaps.

## Search Method

We reviewed the literature for studies on abdominal pain in primary chronic constipation. This review is a narrative synthesis organized around the following research questions before Nov 2024:

1. What are the possible mechanisms or underlying contributors to developing abdominal pain in FC/IBS-C?
2. What are the research gaps in the pathophysiology?
3. What are the available management strategies for abdominal pain in FC/IBS-C?
4. What are the clinical gaps in current therapeutic approaches?
5. What are the possible future research priorities in diagnosis and treatment?

The search was conducted in the Ovid database using the following search terms: (abdominal pain OR pain) AND (constipation). An English language restriction was applied. Basic research articles, clinical trials, and review articles were considered. Clinical case reports, expert opinions, or review letters were excluded. Three reviewers (JY-L, QQ-X, and SJ-X) selected and reviewed the literature. The reviewers conducted an initial screening of titles and abstracts of published articles and reviewed full articles to assess each study's eligibility for inclusion. A study was included if it was considered likely to provide valid and useful information and met the research questions listed above. Disagreements were resolved through discussion.

### Pathophysiological Mechanisms of Abdominal Pain in IBS-C and FC

The pathophysiology of abdominal pain includes a painful stimulus and/or an abnormal peripheral or central pain response to the perception of a normal or abnormal stimulus. In patients with constipation, abnormal distension can contribute to pain. However, the pain may also be triggered by local stimuli or disturbances in the nervous system, regardless of their direct relation to constipation.

**Fig. 1:**
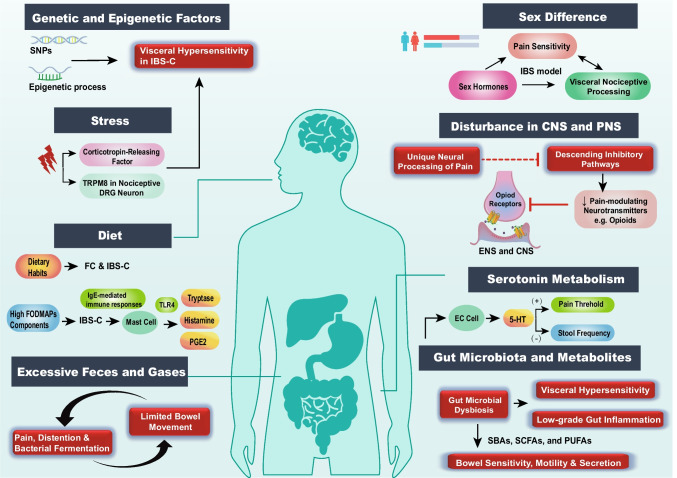
Integrated Pathophysiological Mechanisms of Abdominal Pain in FC and IBS-C. This schematic illustrates the multifactorial pathways contributing to abdominal pain in chronic constipation. Genetic and epigenetic factors contribute to visceral hypersensitivity in IBS-C. Stress responses activate corticotropin-releasing factor and TRPM8 in nociceptive neurons. Dietary habits and components, particularly high-FODMAP foods, trigger mast cell activation. Sex differences modulate pain sensitivity and visceral nociception through hormonal mechanisms. Central and peripheral nervous system alterations affect pain processing via opioid receptors and descending inhibitory pathways. Serotonin metabolism influences both pain threshold and bowel motility in IBS-C. Gut microbiota dysbiosis contributes to low-grade inflammation and visceral hypersensitivity, further affecting bowel sensitivity, motility, and secretion. Restricted bowel movement, pain and/or distention sensation influence each other via accumulated feces and gas, perpetuating a cycle of visceral discomfort. Understanding these interconnected pathways provides insights for therapeutic targets. FC, functional constipation; IBS-C, constipation-predominant irritable bowel syndrome; SNPs, single nucleotide polymorphisms; TRPM8, transient receptor potential melastatin 8; DRG, dorsal root ganglia; FODMAPs, fermentable oligosaccharides, disaccharides, monosaccharides, and polyols; TLR4, toll-like receptor 4; PGE2, prostaglandin E2; CNS, central nervous system; PNS, peripheral nervous system; ENS, enteric nervous system; EC, enterochromaffin; 5-HT, serotonin; SBAs, secondary bile acids; SCFAs, short-chain fatty acids; PUFAs, polyunsaturated fatty acids

The pathophysiology of abdominal pain in IBS-C and FC is complex and multifactorial, involving a variety of crosstalk mechanisms that contribute to the severity and recurrent of symptoms. Here, we present an overview (Fig. 1) of the pathophysiological mechanisms underlying abdominal pain, understanding these diverse contributors is crucial for developing effective therapeutic strategies to manage and alleviate the chronic abdominal pain associated with constipation.

### Excessive Feces and Gases

Limited bowel movements in both FC and IBS-C leads to an accumulation of excessive and hard feces in the gut lumen, which results in abnormal distension and contraction, causing feelings of pain and distension [17]. Defecation disorders, including dyssynergia (the puborectalis is spastic) and anismus (the anal sphincter does not relax), could reduce emptying of the left colon which leads to abdominal pain or distension [22]. Promoting defecation, for example by using laxatives [17], may therefore be effective in relieving abdominal pain associated with constipation.

Infrequent passing of stool also increases bacterial fermentation of luminal content in the small and large intestines, producing gases including hydrogen, methane, and carbon dioxide, which may contribute to distension, flatulence, and abdominal pain [23]. Small intestinal bacterial overgrowth (SIBO) may also produce a large amount of gas in the abdomen, and a subtype of SIBO that leads to excessive methane production has been related to constipation [24]. Therefore, addressing bacterial overgrowth and gas production could be a clue in managing abdominal pain associated with constipation.

### Diet

Dietary habits and food components may influence abdominal pain in both FC and IBS-C, but the underlying mechanisms differ between the two conditions.

Irregular meal timing, binge eating, and high-fat diets have been associated with worsening constipation symptoms, which may indirectly contribute to abdominal discomfort in FC patients [25, 26]. There is currently limited direct evidence linking specific foods to abdominal pain in FC, though dietary factors may influence stool consistency and colonic motility, leading to distension-related discomfort [25, 27–33].

Intra-duodenal lipids infusion, has been shown to enhance colorectal hypersensitivity with an increased perception of rectal distension in IBS-C patients as compared to healthy subjects [34]. In addition, IBS patients are more prone to the exacerbation of abdominal pain, bloating and distension caused by both readily fermentable and insoluble fibers [35, 36]. This is consistent with the emerging dietary approach for IBS patients that restricts foods with highly fermentable oligo-, di-, monosaccharides, and polyols (FODMAPs) [37, 38]. Mechanistic studies suggest that food-induced pain in IBS-C may involve dietary antigen-specific IgE-mediated immune responses, triggering mast cell activation and the release of tryptase, histamine, and prostaglandin E2 via TLR4 signaling [39, 40].

While dietary modifications may help alleviate symptoms in both FC and IBS-C, evidence strongly supports dietary triggers in IBS-C-related abdominal pain through visceral hypersensitivity, immune activation, and gut fermentation processes. In contrast, FC-related pain appears more indirectly linked to dietary factors via stool consistency and colonic motility changes. More research is needed to determine whether specific foods directly contribute to pain in FC patients.

### Gut Microbiota and Their Metabolites

Gut microbiota is integral to the maintenance of bowel sensitivity, motility and secretion. Many studies reported gut microbial dysbiosis in both FC and IBS-C patients [41–46]. Compared to healthy subjects, IBS-C patients have a higher level of mucosal Bacteroidetes and a lower level of *Bifidobacterium* in the feces [46], which provides insight into the potential role of gut microbiota in abdominal pain. Patients with visceral hypersensitivity (VH) have also been shown to harbor a dysbiotic gut microbiota [47], and transplanting fecal microbiota from these patients to germ-free mice produced a similar phenotype in the animals. These data provided evidence for dysbiosis as a contributing factor to VH [48]. Some species in the *Lactobacillus* genus, for instance, *L. paracasei *[49], *L. acidophilus *[50], *L. rhamnosus* and *L. plantarum *[51, 52], have been shown to ameliorate abdominal pain in the antibiotics-induced VH mouse model. Although the mechanisms of such analgesic effects are unclear, deficiency or absence of these bacteria may result in VH. However, their direct role in relieving pain in FC or IBS-C remains unverified and requires further research.

Low-grade intestinal inflammation plays a key role in the pathophysiology of visceral pain [53], and gut microbiota can induce gut inflammation by triggering chronic release of pro-inflammatory mediators from the host such as prostaglandin 2, histamine, and cytokines including IL-1β, IL-6, IL-8, IL-10, and TNF-α [54, 55]. These mediators may lead to activation and sensitization of the nociceptors and unpredictable bouts of visceral pain [56]. Furthermore, gut microbiota-derived histamine has been implicated in visceral hyperalgesia through histamine 4 receptor signaling. Klebsiella aerogenes, identified in some IBS patients' microbiota and carrying a histidine decarboxylase gene, is a major producer of histamine [57]. The microbial metabolites related to intestinal sensory, motor and secretory functions including secondary bile acids, short-chain fatty acids and polyunsaturated fatty acids, may also contribute to abdominal pain in in FC and IBS-C, but the mechanisms are still unclear [41, 58, 59].

Both FC and IBS-C are associated with gut microbiota dysbiosis, but microbial-induced inflammation and visceral hypersensitivity appear more pronounced in IBS-C. Further research is essential to elucidate the specific mechanisms by which gut microbiota and their metabolites influence abdominal pain in FC and IBS-C.

### Serotonin Metabolism

The role of serotonin (5-HT) in the gastrointestinal tract has been highlighted to be involved in the mechanisms behind abdominal pain. 5-HT is a monoamine neurotransmitter and predominantly concentrated in the gastrointestinal tract, platelets and central nervous system [60]. About 90% peripheral serotonin is synthesized in enterochromaffin (EC) cells [61] and regulated by rate-limiting enzyme called tryptophan hydroxylase (Tph) [62]. When the gut is mechanically or chemically stimulated, 5-HT is released and activates receptors on both intrinsic and extrinsic nerves. This activation plays a crucial role in regulating peristalsis (the contraction and relaxation of muscles in the gastrointestinal tract), enhancing secretion, and modulating sensory transmission between the gut and the central nervous system [63].

Shekhar et al. [11] found that plasma 5-HT concentration was inversely correlated with stool frequency and directly correlated with pain threshold in IBS-C patients, suggesting that serotonin dysregulation contributes to visceral hypersensitivity and pain perception in IBS-C. However, while serotonin plays a role in colonic motility and stool consistency in FC, its direct involvement in pain modulation is less clear. The study also reported less pronounced pain intensity in FC compared to IBS-C, indicating that serotonin-related pain mechanisms may not be as significant in FC.

Furthermore, gut bacteria can communicate with EC cells and induce altered serotonin secretion through various gut-microbial metabolites [64]. This interaction underscores the complex crosstalk between gut microbiota and serotonin in perturbing gastrointestinal function and pain. However, whether gut-microbial metabolites alleviate gastrointestinal symptoms and abdominal pain in both FC and IBS-C via regulating peripheral serotonin level remains unclear. Understanding these mechanisms is vital for developing targeted therapies to manage abdominal pain in constipation effectively.

### Genetic and Epigenetic Factors

Genetic and epigenetic factors play a significant role in the familial tendency and visceral sensitivity of IBS-C and FC. Both conditions tend to cluster in families due to shared genetic and/or environmental factors [65–67]. Associations between certain single nucleotide polymorphisms (SNPs) and abdominal pain in IBS-C have been reported [68]. Notably, some of these SNPs are in genes related to the key molecular targets related to VH, such as 5-HT [69], cholecystokinin [70], voltage-gated sodium channels [71], catechol-O-methyltransferase [72], cannabinoids [73], and transient receptor potential channels [32]. Evidence for genetic contributions to pain perception in FC is limited.

Epigenetic processes, as a bridge between environment and genes, have also been associated with VH. For instance, an increase in DNA methylation in genes, such as glutathione S-transferases mu 5 and tubulin polymerization promoting protein, has been found in IBS patients [68]. In addition, differential expression of non-coding RNA (microRNA and long non-coding RNA) including microRNA-24, microRNA-16 and microRNA-103, have been associated with increased visceral sensitivity in IBS patients, [74, 75], but their relevance to FC remains unclear [68]. These insights indicate the interplay between inherited factors and environmental influences in the development of visceral sensitivity in IBS-C.

### Stress

Multiple epidemiological studies have implicated that stress, both psychosocial and/or physical, is a trigger of onset or exacerbation of abdominal pain and bowel dysfunction, particularly in IBS-C [76, 77]. In IBS-C patients, acute mental and acoustic stress slow intestinal motility and increase the colonic spike-burst activity with higher levels of the vital stress hormone corticotropin-releasing factor (CRF). Excessive production of CRF may lead to bowel dysfunction and VH [78–81]. A recent study suggests that acute psychological stress may enhance the function of transient receptor potential melastatin 8 (TRPM8) in the nociceptive dorsal root ganglion (DRG) neurons and induce VH [82]. The relationship between stress and VH has been comprehensively reviewed by *Larauche* et al. [77]. These finding highlights the potential of stress management in treating abdominal pain in IBS-C. However, the relationship between stress and pain in FC is less well defined, as most studies have focused on IBS models rather than FC-specific mechanisms.

### Sex Diffrence

The prevalence of IBS-C and FC is higher in women than men [1, 44, 83], which is consistent with women being more susceptible to stress-related disorders and other comorbid diseases such as fibromyalgia and migraine [84, 85]. Importantly, it is reported that women are at a higher risk than men of developing pain-related disorders and increased sensitivity to induced visceral and somatic pain [85]. James R. Bayrer et al. [86] reported EC cell-mucosal afferent signaling had a different contribution in female compared to male mice, reflecting a higher tonic input of this circuit in females. The higher pain sensitivity in women may be due to sex-specific differences in: 1) ascending pain transmission pathways; 2) descending pain modulation pathways; and/or 3) psychological phenomena that affect pain [87]. These differences in central and peripheral nociceptive pathways indicate that female IBS-C and FC patients may be at a higher risk for chronic abdominal pain than their male counterparts. Data from animal studies (IBS model) provided some mechanistic insights into the relationship between sex and VH: sex hormones were significant contributors to the stimulation of VH in animal models [88]; the reversal of VH by ovariectomy in a female rodent modal established causality between ovarian hormones and visceral sensitivity [89]. A functional MRI study further suggested that estrogen can alter visceral nociceptive processing in the brain following an acute stressor in rats [90]. Together, in IBS-C, hormonal fluctuations may contribute to symptom variability, but whether these mechanisms extend to pain perception in FC remains uncertain.

### Disturbance in the Central Nervous System

Abdominal pain in IBS-C is strongly associated with altered central pain processing. Neuroimaging studies indicate that patients with chronic abdominal pain exhibit increased activity in brain regions responsible for pain perception, emotional regulation, and autonomic responses [91]. However, the extent to which central pain modulation contributes to abdominal pain in FC remains unclear.

Neural processing pathways for visceral pain have been characterized in some patients with IBS-C. Guleria et al. [92] suggested that the activation of brain areas that control emotional motivation, balance of emotions, and autonomic responses to pain in IBS-C patients differed from that in patients with the other IBS subtypes. Wilder-Smith et al. [93] indicated that comparing to other subtypes of IBS patients and healthy subjects, only IBS-C patients showed significant activation in the amygdala, hippocampus and thalamus, and in the cingulate gyrus and prefrontal cortex during heterotopic stimulation. This suggests that IBS-C patients experience a unique neural processing of pain that may be linked to their specific clinical phenotype. Furthermore, descending inhibitory pathways, which modulate the visceral pain signal by releasing neurotransmitters such as opioids, noradrenaline, dopamine and 5-HT [94], are down-regulated in IBS patients as indicated by an increased somatic sensitivity [95]. This down-regulation implies a reduced capacity to inhibit pain signals, potentially exacerbating chronic pain experiences in IBS patients.

### Disturbance in the Peripheral Nervous System

Beyond central mechanisms, peripheral nerve dysfunction may contribute to visceral pain in constipation-related disorders. Both stress-induced changes and direct alterations in peripheral nociceptive pathways have been implicated in pain modulation in IBS-C, with limited data available for FC. John H. Winston et al. [96] demonstrated heterotypic chronic stress (HeCS) could induce visceral hypersensitivity and sensitize colon-specific dorsal root ganglion (DRG) neurons through improving expression of nerve growth factor (NGF) in colonic muscularis externa and mucosa/submucosa. This indicates that stress can directly impact the sensitivity of peripheral neurons, contributing to the heightened pain perception in IBS-C patients. The enteric nervous system (ENS), named “second-brain”, is composed by thousands of small ganglia interconnected by neural fibers and arranged in two plexuses [97]. Opioid receptors located in both central nervous system (CNS) and the enteric nervous system (ENS) were associated with visceral hypersensitivity and gastrointestinal motility control in IBS patients [98], indicating they could be a potential treatment target for abdominal pain in IBS. Recent research also highlights that the expression of Piezo-type mechanosensitive ion channel component 2 (Piezo2) in colonic epithelial cells correlates with pain sensation in IBS. Piezo2, expressed in TRPV1-lineage nociceptors innervating the colon, contributes significantly to visceral mechanosensitive and nociception under both normal and pathological conditions [99–101] Additionally, TRPV1-expressed visceral neurons play a crucial role in chronic visceral pain through microglial activation in the spinal cord, mediated by purinergic signaling involving neuronal ATP and microglial P2Y12 receptors [102]. These findings highlight the intricate interplay between mechanosensitive and heat-sensitive channels in the PNS and CNS, suggesting Piezo2 as a potential therapeutic target for visceral pain treatment.

In summary, the pathophysiology of abdominal pain in chronic constipation involves a complex interplay of mechanical, microbial, neuroendocrine, and genetic-epigenetic mechanisms. Accumulated fecal matter and gas induce colonic distension and dyssynergia. Dietary habits exacerbate pain in FC via altered motility, while immune activation amplify food-triggered pain in IBS-C. Gut microbiota dysbiosis and metabolites (e.g., histamine, SCFAs) modulate visceral sensitivity and low-grade inflammation, affecting bowel motility, sensitivity and secretion. Serotonin imbalances disrupt motility and pain signaling, with stronger links to IBS-C. Though FC and IBS-C both show familial tendency, genetic/epigenetic variants (e.g., SNPs in pain-related genes, microRNA dysregulation) and stress-induced corticotropin release further heighten susceptibility, particularly in IBS-C. Sex differences, influenced by hormonal fluctuations and neural circuit variability, predispose women to heightened pain perception. Central and peripheral nervous system disturbances, including altered brain processing, downregulated pain inhibition, and mechanosensitive ion channels (e.g., Piezo2, TRPV1), underpin chronic pain in IBS-C, while their role in FC remains unclear. Current evidence predominantly derives from IBS patients or animal models, with limited research specifically addressing pain mechanisms in FC. Further research is needed to clarify whether FC shares similar pain pathways with IBS-C or involves distinct mechanisms. Elucidation on these pathophysiological mechanisms provide a comprehensive framework for further research and potential therapeutic advancements.

### Management for Abdominal Pain in Patients with Constipation

Treatment for abdominal pain in patients with constipation is a challenge for physicians due to dynamic change of symptoms and complexity of the pathophysiology. A combination treatment is usually prescribed to achieve optimal patient outcomes in the real-world practice [103]. While many therapies focus on improving bowel function, fewer directly address visceral hypersensitivity and pain modulation. A multimodal approach targeting gut motility, sensory processing, and neuroimmune regulation is essential.

### Pharmacological Approaches

Anti-spasmodics, including dicyclomine, hyoscyamine, and hyoscine, may reduce abdominal pain by relaxing intestinal smooth muscle, but can worsen constipation and lack strong evidence for IBS-C pain relief [104, 105]. Peppermint oil has shown potential analgesic effects via calcium channel blockade and TRPM8 modulation, though its efficacy in constipation-related pain remains uncertain [106]. Laxatives are commonly used to treat constipation alone [17], and a systematic review indicates that use of laxation in constipation can attenuate abdominal pain [107], possibly by eliminating excessive stool and gases in the colon. 5-HT_4_ agonists (Tegaserod, prucalopride): Tegaserod reduces abdominal pain in IBS-C by modulating gut motility and visceral sensitivity, but its use is restricted due to cardiovascular risks [108, 109]. Prucalopride, while improving bowel function, has limited evidence for pain relief [110]. Guanylate Cyclase-C Agonists (Linaclotide, Plecanatide): These agents reduce visceral hypersensitivity via cGMP signaling and provide pain relief in IBS-C, but diarrhea is the most common adverse effect limiting its use in certain patients [111–113]. Sodium/hydrogen exchanger 3 (NHE3) inhibitor (Tenapanor): Shown to reduce abdominal pain and bloating in IBS-C by modulating sodium transport and visceral sensitivity, though long-term safety remains under investigation [114–116]. Chloride Channel Activator (Lubiprostone): it can improve pain and stool consistency within one month, though its long-term benefits over placebo are unclear [117, 118]. Adverse effects include nausea (8%−19%) and diarrhea (6%−14%), limiting its tolerability [119].

### Psychological Interventions

CNS dysfunction contributes to abdominal pain in IBS-C, making neuromodulators and psychological therapies potential treatment options. Antidepressants, including tricyclic antidepressants (TCAs), selective serotonin reuptake inhibitors (SSRIs) and serotonin and norepinephrine re-uptake inhibitors (SNRIs), show superior pain relief in IBS-C compared to placebo [120], with SSRIs also improving motility [121]. However, their use is limited by side effects (drowsiness, constipation), risk of dependency, and individual variability in drug response. Psychological therapies such as cognitive-behavior therapy and gut-directed hypnotherapy also appears to be beneficial in reducing abdominal pain as they aim to address the psychological aspects of pain and improve coping mechanisms, but more high-quality evidence is needed [120].

### Microbiota Modulator and Complementary Medicine

Probiotics have gained attention as a potential treatment for abdominal pain and constipation; however, the use of probiotics has yielded inconsistent findings, primarily due to variations in combinations of strains used and differences in study endpoints. These inconsistencies make it challenging to draw definitive conclusions regarding the efficacy of probiotics in the treatment of abdominal pain associated with constipation [122]. While gut microbiota's role in pain modulation is recognized, specific mechanisms remain unclear. Similar challenges exist with complementary therapies, where acupuncture and certain Chinese herbal formulations show potential benefits in IBS, but variability in composition and lack of standardization hinder adoption [123, 124]. However, the variability in herbal compositions and the lack of standardization pose significant challenges to their widespread adoption. High-quality studies are needed to confirm their effectiveness in constipation related pain management.

In summary, managing abdominal pain in constipation requires a multimodal approach addressing gut motility, visceral hypersensitivity, and neuroimmune dysregulation. Antispasmodics (e.g., hyoscyamine) and peppermint oil alleviate abnormal contractions but lack robust evidence in constipation-related pain, while laxatives improve stool passage but show limited direct analgesic efficacy. Targeted agents like 5-HT4 agonists (tegaserod, prucalopride), guanylate cyclase-C agonists (linaclotide), and NHE3 inhibitors (tenapanor) modulate motility and pain signaling but face limitations such as side effects (e.g., diarrhea, cardiovascular risks) or insufficient long-term data. Psychological interventions, including antidepressants and cognitive-behavioral therapy, address central pain processing but are hindered by adverse effects and variable responses. Microbiota modulators (probiotics) and complementary medicine show potential but lack consistency due to strain variability and unstandardized formulations. There is still a clinical and research gap in this context. A tailored, integrated, patient-centred treatment is essential to optimize pain management in constipation.

### Future Directions and Conclusion

In conclusion, managing abdominal pain in IBS-C and FC remains a formidable challenge due to its complex and multifactorial pathophysiology. To enhance diagnosis and treatment, future research should focus on elucidating the underlying mechanisms and identifying reliable biomarkers. Longitudinal studies that track changes in symptoms over time and large-scale human cohorts to collect detailed multimodal data are promising for capturing the dynamic nature of symptoms and explaining inter-individual variations [125]. Multi-omics and metadata studies may simultaneously analyze multiple biological variables alongside clinical data, enabling the identification of complex interaction patterns and novel biomarker signatures of abdominal pain in IBS-C and FC. Employing advanced analytical tools, such as machine learning, could uncover associations between abdominal pain and IBS-C and FC, explaining temporal changes in pain, and identifying risk factors. By integrating these insights, we can move towards more effective and tailored management strategies, ultimately improving patient outcomes and quality of life.

## Key Reference

- Sperber AD, Bangdiwala SI, Drossman DA, Ghoshal UC, Simren M, Tack J, et al. Worldwide Prevalence and Burden of Functional Gastrointestinal Disorders, Results of Rome Foundation Global Study. Gastroenterology. 2021;160(1):99–114.e3.
  - Comprehensive analysis of the global prevalence and burden of functional gastrointestinal disorders including IBS-C and FC. This serves as a foundational reference for understanding the widespread nature and significant burden of abdominal pain and chronic constipation.
- Bharucha AE, Lacy BE. Mechanisms, Evaluation, and Management of Chronic Constipation. Gastroenterology. 2020;158(5):1232–49.e3.
  - Comprehensive summary of the mechanisms underlying chronic constipation and its management. It is valuable for understanding the multifactorial nature of chronic constipation, including motility disorders and sensory dysfunctions, which are directly relevant to the manuscript's focus on abdominal pain mechanisms.
- Shekhar C, Monaghan PJ, Morris J, Issa B, Whorwell PJ, Keevil B, et al. Rome III functional constipation and irritable bowel syndrome with constipation are similar disorders within a spectrum of sensitization, regulated by serotonin. Gastroenterology. 2013;145(4):749–57; quiz e13-4.
  - This article explores the similarities between Rome III functional constipation and IBS-C, proposing that they are part of a spectrum of disorders regulated by serotonin. It provides insights into the shared pathophysiological mechanisms, emphasizing the role of serotonin in these conditions.
- Lacy BE, Pimentel M, Brenner DM, Chey WD, Keefer LA, Long MD, et al. ACG Clinical Guideline: Management of Irritable Bowel Syndrome. Am J Gastroenterol. 2021;116(1):17–44.
  - This clinical guideline from the American College of Gastroenterology provides evidence-based recommendations for the management of IBS-C, which is essential for understanding the current practice strategies and limitations.

## Data Availability

No datasets were generated or analysed during the current study.
